# Granger causality revisited

**DOI:** 10.1016/j.neuroimage.2014.06.062

**Published:** 2014-11-01

**Authors:** Karl J. Friston, André M. Bastos, Ashwini Oswal, Bernadette van Wijk, Craig Richter, Vladimir Litvak

**Affiliations:** a The Wellcome Trust Centre for Neuroimaging, University College London, Queen Square, London WC1N 3BG, UK; b Center for Neuroscience and Center for Mind and Brain, University of California-Davis, Davis, CA 95618, USA; c Ernst Strüngmann Institute in Cooperation with Max Planck Society, Deutschordenstraße 46, 60528 Frankfurt, Germany

**Keywords:** Granger causality, Dynamic causal modelling, Effective connectivity, Functional connectivity, Dynamics, Cross spectra, Neurophysiology

## Abstract

This technical paper offers a critical re-evaluation of (spectral) Granger causality measures in the analysis of biological timeseries. Using realistic (neural mass) models of coupled neuronal dynamics, we evaluate the robustness of parametric and nonparametric Granger causality. Starting from a broad class of generative (state-space) models of neuronal dynamics, we show how their Volterra kernels prescribe the second-order statistics of their response to random fluctuations; characterised in terms of cross-spectral density, cross-covariance, autoregressive coefficients and directed transfer functions. These quantities in turn specify Granger causality — providing a direct (analytic) link between the parameters of a generative model and the expected Granger causality. We use this link to show that Granger causality measures based upon autoregressive models can become unreliable when the underlying dynamics is dominated by slow (unstable) modes — as quantified by the principal Lyapunov exponent. However, nonparametric measures based on causal spectral factors are robust to dynamical instability. We then demonstrate how both parametric and nonparametric spectral causality measures can become unreliable in the presence of measurement noise. Finally, we show that this problem can be finessed by deriving spectral causality measures from Volterra kernels, estimated using dynamic causal modelling.

## Highlights

• This paper describes the evaluation of expected Granger causality measures.
• It uses these measures to quantify problems with dynamical instability and noise.
• These problems are resolved by basing Granger measures on DCM estimates.

## Introduction

This paper concerns the application of Granger causality to biological timeseries; in particular, the analysis of electrophysiological data using (complex) cross spectra (Granger, 1969). Our focus is on spectral causality measures of the sort introduced by Geweke (Dhamala et al., 2008, Geweke, 1982, Nedungadi et al., 2009) and their relationship to dynamic causal modelling (Friston et al., 2013).

We first show how Granger causality can be derived from the parameters of a state-space model of coupled neuronal dynamics — and establish that Granger causality correctly reports the true direction of coupling, under low levels of measurement noise. We then consider two cases where Granger causality provides unreliable estimates of the underlying effective connectivity. First, when the dynamics generating timeseries data contain slow (unstable) modes, recovering the autoregressive coefficients used in parametric Granger causality becomes an ill-posed problem. This is important because unstable modes are ubiquitous in biological systems; for example, in systems with scale-free characteristics (such as power laws) or dynamics associated with self-organised criticality (Bullmore et al., 2001, Shin and Kim, 2006, Stam and de Bruin, 2004). This problem can be resolved by using nonparametric^1^ Granger causality that eschews autoregressive parameterisations (Dhamala et al., 2008, Nedungadi et al., 2009). However, both parametric and nonparametric Granger causality can fail in the context of measurement noise. This is an established shortcoming of Granger causality (Newbold, 1978) but becomes particularly acute with noisy electrophysiological recordings (Nalatore et al., 2007). Having characterised these two problems, we consider a solution using Granger causal measures based on the posterior parameter estimates of dynamic causal modelling.

Unlike Granger causality, dynamic causal modelling uses an explicit model of the dynamics generating data — usually cast in terms of a state-space model with hidden states. This is crucial because DCM mandates an explicit parameterisation of both random *fluctuations* perturbing hidden states and measurement *noise*. In contrast, the *innovations* assumed to underlie random effects in autoregressive processes do not make this distinction — and have to assume that the data are noiseless (Nalatore et al., 2007). However, in principle, the effects of measurement noise can be removed using DCM and the resulting Granger causal measures can be derived from the estimated model parameters furnished by model inversion. These points are described below using standard (analytic) results and numerical simulations. This treatment offers a way forward for Granger causality in the context of measurement noise and long-range correlations; however, there are many outstanding issues in the setting of DCM that we will return to in the discussion.

This paper comprises three sections. In the first, we review the relationship between state-space models and various characterisations of their second-order behaviour, such as coherence and spectral Granger causality. This section describes the particular state-space model used for subsequent simulations. This model is based upon a standard neural mass model that is part of the suite of models used in the dynamic causal modelling of electromagnetic data (David et al., 2006, Friston et al., 2012, Moran et al., 2008). The section concludes by showing that – in the absence of noise and with well-behaved (stable) dynamics – expected Granger causal measures are accurate and properly reflect the underlying causal architecture. In the second section, we vary some parameters of the model (and measurement noise) to illustrate the conditions under which Granger causality fails. This section focuses on failures due to critical (unstable) dynamics and measurement noise using heuristic proofs and numerical simulations. The final section shows that, in principle, Bayesian model inversion with DCM dissolves the problems identified in the previous section; thereby providing veridical Granger causal measures in frequency space.

## Models and measures of causality in dynamic systems

The purpose of this section is to clarify the straightforward relationships between spectral descriptions of data and the processes generating those data. This is important because if we know – or can estimate – the parameters of the generative process, then one can derive expected measures – such as cross-covariance functions, complex cross spectra, autoregressive coefficients and directed transfer functions – analytically. In other words, measures that are typically used to characterise observed data can be regarded as samples from a probability distribution over functions, whose expectation is known. This means that one can examine the expected behaviour of normalised measures – like cross-correlation functions and spectral Granger causality – as an explicit function of the parameters of the underlying generative process. We will use this fact to see how Granger causality behaves under different parameters of a neural mass model generating electrophysiological observations — and different parameters of measurement noise.

In what follows, we use *functional connectivity* to denote a statistical dependence between two measurements and *effective connectivity* to denote a causal influence among hidden (neuronal) states that produce functional connectivity. By definition, effective connectivity is directed, while directed functional connectivity appeals to constraints on the parameterisation of statistical dependencies that preclude non-causal dependencies. Because we will be discussing state-space and autoregressive formulations, we will also make a distinction between *fluctuations* that drive hidden states and *innovations* that underlie autoregressive dependencies among observations. Innovations are a fictive construct (effectively a mixture of fluctuations and measurement noise) that induce an autoregressive form for statistical dependencies over time. Fourier transforms will be denoted by **F**[·], expectations by **E**[·] convolution operators by * and Kronecker tensor products by ⊗. Variables with a ~ denote (usually Toeplitz) matrices whose columns contain (lagged) functions of time and † means conjugate transpose.

Table 1a provides the basic form of the generative models that we will consider. This form is based on (stochastic and delay differential) equations of motion and a static mapping to observations. Any system of this sort has an equivalent Volterra series expansion that can be summarised in terms of its first order Volterra kernels. These kernels can be thought of as an impulse response to each source of fluctuations.

**Table 1:** This table presents the expressions that relate unnormalised and normalised measures of second-order statistical dependencies among data to the underlying process generating those data. Table 1a specifies the generative (state-space) model in terms of stochastic differential equations of motion and a static nonlinear observer function. The random fluctuations that perturb the motion of hidden (neuronal) states and the observation noise are characterised in terms of their second-order statistics; namely their covariance or spectral density. These state-space models can be formulated in terms of a convolution of the fluctuations, where the (first order Volterra) convolution kernels are a function of the model parameters. Table 1b shows how these kernels can generate any characterisation of the ensuing dependencies among the data – as cross-covariance functions of lag or time, cross spectral density functions of frequency and autoregressive formulations – in terms of autoregression coefficients and associated directed transfer functions. The expressions have been simplified and organised to illustrate the formal symmetry among the relationships. The key point to take from these expressions is that any characterisation can be derived analytically from any other using Fourier transforms **F**[·], expectations **E**[·] convolution operators * and Kronecker tensor products ⊗. Variables with a ~ denote matrices whose columns contain lagged functions of time and † denotes the conjugate transpose. Table 1c provides standardised versions of the second order statistics in Table 1b. These include the cross-correlation function, coherence, Geweke Granger causality and the normalised (Kaminski) directed transfer functions. These results mean that we can generate the expected Granger causality from the parameters of any generative model in exactly the same way that any other data feature can be generated. Note that in going from a parameterised generative model to the second-order statistics, there is no return. In other words, although second-order statistics can be generated given the model parameters, model parameters cannot be derived from second-order statistics. This is the (inverse) problem solved by DCM for complex cross spectra — that requires a generative model.

a: state-space model		
State space model	Random fluctuations	Convolution kernels
$\begin{array}{c} \dot{x}\left(t\right)=f\left(x,\theta \right)+v\left(t\right)\\ y\left(t\right)=g\left(x,\theta \right)+w\left(t\right) \end{array}$	$\begin{array}{c} E[v\left(t\right)\cdot v(t-\tau ){}^{T}]=\Sigma_{v}(\tau ,\theta )\\ E[w\left(t\right)\cdot w(t-\tau ){}^{T}]=\Sigma_{w}(\tau ,\theta ) \end{array}$	$\begin{array}{c} y\left(t\right)=k(\tau )*v(t)+w(t)\\ k\left(\tau \right)=\nabla_{x}g(x_{0},\theta )\cdot \exp\left(\tau \cdot \nabla_{x}f\left(x_{0},\theta \right)\right) \end{array}$


b: second-order dependencies				
	Cross covariance Σ(*t*)	Cross spectral density *g*(*ω*)	Autoregression coefficients *a*	Directed transfer functions *S*(*ω*)
Cross covariance Σ(*t*)	*Σ*(*t*) = *k*(*τ*) ∗ *Σ*_*v*_ ∗ *k*(*τ*) + *Σ*_*w*_	*Σ*(*t*) = **F**^− 1^[*g*(*ω*)]	$C={\left(I-\tilde{a}\right)}^{-1}\left(\sum_{z}⊗I\right){\left(I-\tilde{a}\right)}^{-T}$	*Σ*(*t*) ∝ **F**^− 1^[*S*(*ω*) ⋅ *Σ*_*z*_ ⋅ *S*(*ω*)^†^]
Cross spectral density *g*(*ω*)	*g*(*ω*) = **F**[*Σ*(*τ*)]	$\begin{array}{l} g\left(\omega \right)=K\left(\omega \right)\cdot g_{v}\cdot K{\left(\omega \right)}^{†}+g_{w}\\ K\left(\omega \right)=F\left[k\left(\tau \right)\right] \end{array}$	$\begin{array}{l} g\left(\omega \right)=S\left(\omega \right)\cdot \Sigma_{z}\cdot S{\left(\omega \right)}^{†}\\ S\left(\omega \right)={\left(I-F\left[a\right]\right)}^{-1} \end{array}$	$\begin{array}{c} g\left(\omega \right)\propto S\left(\omega \right)\cdot \Sigma_{z}\cdot S{\left(\omega \right)}^{†}\\ =\Psi \left(\omega \right)\cdot \Psi {\left(\omega \right)}^{†} \end{array}$
Autoregression coefficients *a*	$a=C^{-1}\tilde{\Sigma }$	$\begin{array}{c} a=C^{-1}\tilde{\Sigma }\\ \Sigma \left(\tau \right)=F^{-1}\left[g\left(\omega \right)\right] \end{array}$	$\begin{array}{c} y=\tilde{y}\cdot a+z\Rightarrow \\ a=E{\left[{\tilde{y}}^{T}\tilde{y}\right]}^{-1}E\left[{\tilde{y}}^{T}y\right]\\ \Sigma_{z}=E\left[z^{T}z\right]\propto \psi \left(0\right)\cdot \psi {\left(0\right)}^{†}\\ =\Sigma \left(0\right)-{\tilde{\Sigma }}^{T}C^{-1}\tilde{\Sigma } \end{array}$	$\begin{array}{c} a=F^{-1}\left[A\left(\omega \right)\right]\\ A\left(\omega \right)=I-S{\left(\omega \right)}^{-1}\\ S\left(\omega \right)=\Psi \left(\omega \right)\cdot \psi {\left(0\right)}^{-1}\\ \psi =F^{-1}\left[\Psi \left(\omega \right)\right] \end{array}$
Directed transfer functions *S*(*ω*)	$S\left(\omega \right)={\left(I-F\left[C^{-1}\tilde{\Sigma }\right]\right)}^{-1}$	$\begin{array}{c} S\left(\omega \right)={\left(I-F\left[C^{-1}\tilde{\Sigma }\right]\right)}^{-1}\\ \Sigma \left(\tau \right)=F^{-1}\left[g\left(\omega \right)\right] \end{array}$	$\begin{array}{c} S\left(\omega \right)={\left(I-A\left(\omega \right)\right)}^{-1}\\ A\left(\omega \right)=F\left[a\right] \end{array}$	$\begin{array}{c} Y\left(\omega \right)=A\left(\omega \right)\cdot Y\left(\omega \right)+Z\left(\omega \right)\\ =S\left(\omega \right)\cdot Z\left(\omega \right) \end{array}$


c: normalised measures			
Cross correlation	Coherence	Granger causality	Normalised directed transfer functions
$\rho_{\mathrm{ij}}\left(\tau \right)=\frac{\sum_{\mathrm{ij}}\left(\tau \right)}{\sqrt{\sum_{\mathrm{ii}}\left(0\right)\cdot \sum_{\mathrm{jj}}\left(0\right)}}$	$\gamma_{\mathrm{ij}}\left(\omega \right)=\frac{{\left|g_{\mathrm{ij}}\left(\omega \right)\right|}^{2}}{g_{\mathrm{ii}}\left(\omega \right)g_{\mathrm{jj}}\left(\omega \right)}$	$G_{\mathrm{ij}}\left(\omega \right)=-\ln\left(1-\left(\Sigma_{\mathrm{zjj}}-\frac{\Sigma_{\mathrm{zij}}^{2}}{\Sigma_{\mathrm{zii}}}\right)\frac{{\left|S_{\mathrm{ij}}\left(\omega \right)\right|}^{2}}{g_{\mathrm{ii}}\left(\omega \right)}\right)$	$D_{\mathrm{ij}}\left(\omega \right)=\frac{\left|S_{\mathrm{ij}}\left(\omega \right)\right|}{\left|S_{\mathrm{ii}}\left(\omega \right)\right|}$

$$\begin{array}{cccc} C=E\left[{\tilde{y}}^{T}\tilde{y}\right]:C_{\mathrm{ij}}=\left[\begin{array}{ccc} \Sigma_{\mathrm{ij}}\left(0\right) & \cdots & \Sigma_{\mathrm{ij}}\left(-p\right)\\ \vdots & \ddots & \vdots \\ \Sigma_{\mathrm{ij}}\left(p\right) & \cdots & \Sigma_{\mathrm{ij}}\left(0\right) \end{array}\right] & \tilde{\Sigma }=E\left[{\tilde{y}}^{T}y\right]:{\tilde{\Sigma }}_{\mathrm{ij}}=\left[\begin{array}{c} \Sigma_{\mathrm{ij}}\left(1\right)\\ \vdots \\ \Sigma_{\mathrm{ij}}\left(p+1\right) \end{array}\right] & \tilde{y}=\left[\begin{array}{cccc} 0 & & & \\ y_{11} & 0 & & \\ y_{12} & y_{11} & 0 & \\ \vdots & & \ddots & \ddots \end{array}\begin{array}{cccc} 0 & & & \\ y_{21} & 0 & & \\ y_{22} & y_{21} & 0 & \\ \vdots & & \ddots & \ddots \end{array}\ldots \right] & {\tilde{a}}_{\mathrm{ij}}=\left[\begin{array}{cccc} 0 & & & \\ a_{\mathrm{ij}1} & 0 & & \\ a_{\mathrm{ij}2} & a_{\mathrm{ij}} & 0 & \\ \vdots & & \ddots & \ddots \end{array}\right] \end{array}$$

Table 1b shows how various measures of spectral power or variance (second-order statistics) can be derived from the kernels — and from each other. This table is arranged so that the representations of second-order statistics listed against the columns can be derived from the representations over the rows (see the glossary of variables that accompanies this table). For example, cross spectral density is the Fourier transform of the cross covariance function. The entries along the leading diagonal define the models of (linear) dependency upon which these characterisations are based. The odd and even columns pertain to functions of time and frequency respectively, where the Fourier transforms of the kernels are known as transfer functions, which we will refer to as *modulation transfer functions* to distinguish them from *directed transfer functions* (see below). The modulation transfer functions in turn specify the cross spectral density and the cross-covariance function. Note that deriving second-order measures from the (effective connectivity) parameters of the generative process is a one-way street. One cannot recover the parameters from the kernels — in the sense that the mapping from parameters to kernels is not bijective (there are many combinations of parameters that produce the same kernels).

Second-order measures like cross-covariance and spectral density functions do not speak to directed functional connectivity because they do not appeal to any temporal precedence constraints — they simply reflect (non-causal) statistical dependence. In contrast, characterisations based upon autoregressive processes and causal spectral factors are measures of *directed* statistical dependencies because they preclude non-causal influences. Autoregressive formulations do this by conditioning the current observation on previous observations (but not future observations), while causal spectral factors correspond to filters (transfer functions), whose Fourier transforms have zero values at future time points. These are also known as minimum phase filters. Parametric Granger causality uses autoregressive formulations, while nonparametric measures are generally based on (Wilson–Burg) spectral matrix factorisation (Dhamala et al., 2008, Nedungadi et al., 2009). Intuitively, this uses Newton's method to find a square root (or factor) under the constraint that the Fourier transform of the factor is causal or a minimum phase filter (Fomel et al., 2003). Once the cross spectral density has been factorised, the instantaneous part of the filter is removed — and becomes an estimate of the cross covariance of the innovations. The expressions in Table 1b use standard linear algebra results based upon Wiener–Khinchin theorem, the Yule–Walker relationships and (Wilson–Burg) spectral factorisation to show the relationships between different approaches to characterising second-order dependencies between time series.

Note above the difference between the modulation transfer function *K*(*ω*), the directed transfer function *S*(*ω*) and the causal spectral factors *Ψ*(*ω*). These all play similar roles as filters or transfer functions but are distinct characterisations: the modulation transfer function is applied to the fluctuations to produce the observations, whereas the (unnormalised) directed transfer function is applied to the innovations. The directed transfer function only becomes the modulation transfer function – that mediates causal influences – in the absence of measurement noise. Finally, the causal spectral factors include both instantaneous influences and those embodied by directed transfer functions. Table 1c lists the normalised or standardised versions of non-causal (cross-covariance and spectral) and causal (directed transfer) functions. These are commonly used as the basis of inference about undirected and directed functional connectivity respectively (Friston et al., 2013).

**Fig. 1:**
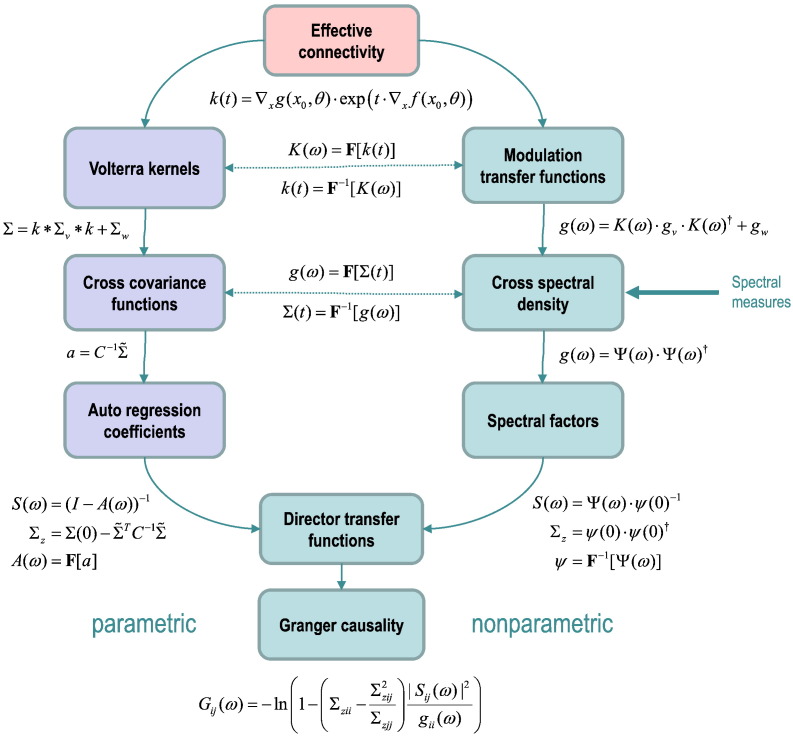
This schematic illustrates the different routes one could take – using the equations in Table 1 – to derive (spectral) Granger causality measures from the (effective connectivity) parameters of a model — or indeed empirical measures of cross spectral density. The key point made by this schematic is the distinction between parametric and nonparametric spectral causality measures. These both rest upon the proportion of variance explained, implicit in the directed transfer functions; however, in the parametric form, the transfer functions are based upon an autoregression model. In contrast, the nonparametric approach uses spectral matrix factorisation, under the constraint that the spectral factors are causal or minimum phase filters. The boxes in light green indicate spectral characterisations, while the light blue boxes indicate measures in the time domain. See Table 1 and main text for a more detailed explanation of the variables and operators.

Equipped with the expressions in Table 1, one can derive the expected functions that characterise functional connectivity, given the parameters of the underlying state-space model. Furthermore, one can derive any one representation from another. For example, starting from a parameterised state-space model, we can derive the Volterra kernels and resulting cross-covariance functions among observation channels. One can then compute the autoregressive coefficients and directed transfer functions to compute parametric spectral Granger causality. Alternatively, one could take the Fourier transform of the kernels (and the cross-covariance functions of the fluctuations and measurement noise) to produce the expected cross spectrum over observed channels. Using spectral matrix factorisation, one can then identify the nonparametric directed transfer function and associated Granger causality. These two (parametric and nonparametric) routes are illustrated schematically in Fig. 1.

**Fig. 2:**
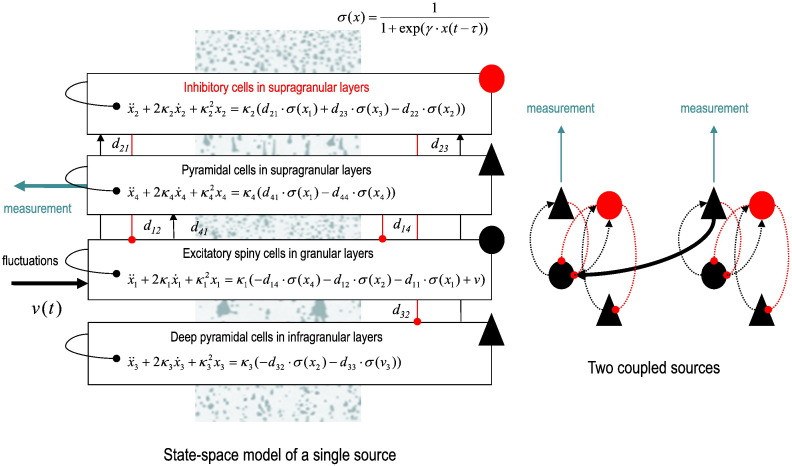
This schematic illustrates the state-space or dynamic causal model that we used to generate expected cross spectra and simulated data. *Left panel*: this shows the differential equations governing the evolution of depolarisation in four populations constituting a single electromagnetic source (of EEG, MEG or LFP measurements). These equations are expressed in terms of second-order differential equations that can be rewritten as pairs of first order equations, which describe postsynaptic currents and depolarisation in each population. These populations are divided into input cells in granular layers of the cortex, inhibitory interneurons and (superficial and deep) principal or pyramidal cell populations that constitute the output populations. The equations of motion are based upon standard convolution models for synaptic transformations, while coupling among populations is mediated by a sigmoid function of (delayed) mean depolarisation. The slope of the sigmoid function corresponds to the intrinsic gain of each population. Intrinsic (within-source) connections couple the different populations, while extrinsic (between-source) connections couple populations from different sources. The extrinsic influences (not shown) enter the equations in the same way as the intrinsic influences but in a laminar specific fashion (as shown in the right panel). *Right panel*: this shows the simple two source architecture used in the current paper. This comprises one lower source that sends forward connections to a higher source (but does not receive reciprocal backward connections). The intrinsic connectivity (dotted lines) and extrinsic connectivity (solid line) conform to the connectivity of the canonical microcircuit and the known laminar specificity of extrinsic connections (Bastos et al., 2012). Excitatory connections are in red and inhibitory connections are in black. Random fluctuations drive the input cells and measurements are based on the depolarisation of superficial pyramidal cells. See Table 2 for a list of key parameters and a brief description.

To illustrate the derivation of expected transfer functions and Granger causality, we will use a standard state-space model from the suite of neural mass models used in the SPM software implementation of dynamic causal modelling. This suite includes neural mass models based upon convolution operators (that model synaptic convolution of presynaptic inputs) or models that are nonlinear in the hidden states based upon conductance models (that model the interaction between voltage and transmembrane conductances). All of these neuronal mass models allow for the coupling of multiple sources, where each source comprises multiple neuronal populations (usually three or four). Fig. 2 shows the canonical microcircuit neural mass model – a convolution model – that we will use in this paper. This particular model has been used previously to characterise things like intrinsic gain control mechanisms in hierarchical visual processing (Brown and Friston, 2012) and impaired top–down connectivity in minimally conscious states (Boly et al., 2011).

Because our focus is on spectral Granger causality, we limit ourselves to a simple bivariate case — with two channels reporting observed depolarisation in two sources. To examine the validity of expected causal measures, we consider the simplest case of a unidirectional (forward) connection from the first to the second source — that is not reciprocated. We wanted to see whether spectral Granger causality could properly discount the backward connections. Fig. 2 details the architecture of this model, with four populations per source and one extrinsic forward connection between the sources. The intrinsic connections couple different populations within a source — here, spiny stellate cells, inhibitory interneurons and superficial and deep pyramidal cells. The equations in the boxes are the equations of motion that constitute the state-space model. Notice that these are delay differential equations because the sigmoid function of presynaptic input operates on the mean depolarisation of the presynaptic source in the recent past — to accommodate axonal conduction delays. Intrinsic (within source) conduction delays are about 1 ms while extrinsic (between source) delays are about 4 to 8 ms.

**Fig. 3:**
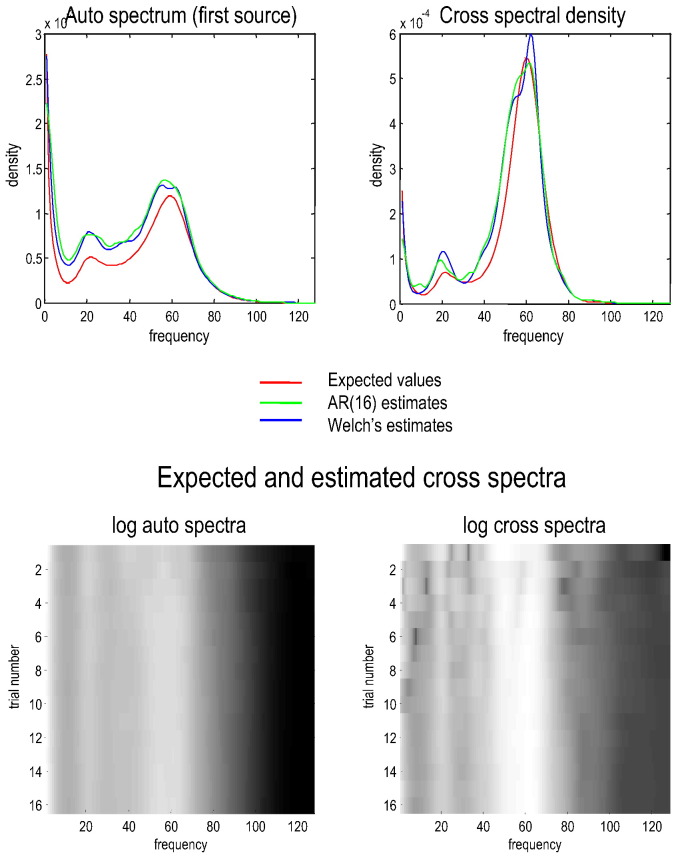
This figure illustrates the convergence of empirical estimates of spectral density averaged over multiple trials. The top row shows the absolute values of the auto (for the first source) and cross spectral density (between the two sources of Fig. 2). The red lines correspond to the expected spectra under the known parameters of the model (the parameters used for characterising spectral measures in subsequent figures). The green and blue lines correspond to empirical estimates based upon 16 epochs of simulated (noisy) data, where each epoch comprised 1024 samples at a sampling rate of 256 Hz. The green lines report the estimates under an AR(16) model, while the blue lines used Welch's periodiogram method, as implemented in Matlab. Both give very similar results. The lower panels show the (absolute value) of the emerging average over 16 trials to show that stable estimates obtain after about eight trials — although many more are generally used in practice to obtain smooth spectral estimates.

We assumed that observed data were generated by the superficial pyramidal cells, with parameterised measurement noise. The (neuronal) fluctuations driving both the spiny stellate cells and the measurement noise had a 1/f or power law form. The amplitude of the measurement noise was suppressed to low values with a log scaling of − 8. In other words, the spectral density of the measurement noise was *g*_*w*_(*ω*) = exp(− 8) ⋅ *ω*^− 1^, where the frequency *ω* range from one to 128 Hz. We used a sampling rate of 256 Hz or a sampling interval of about 4 ms. Using the parameters in Table 2, this pair of sources produces spectra of the sort shown in Fig. 3. The expected spectra (red line) showed clear spectral peaks at beta and gamma frequencies superimposed on a power law form. The blue and green lines show the empirical estimates over 16 (1024 ms) epochs based on simulated data. Appendix A describes how the delay differential equations in Fig. 2 were integrated.

**Table 2:** This table provides the parameter values used for simulations (and prior densities used for subsequent dynamic causal modelling). The left column describes the parameters (corresponding to the equations in Fig. 2). The second column provides the values used to produce the spectra shown in Fig. 3, Fig. 4. The final two columns provide the prior mean and variance for dynamic causal modelling. Note that the variance is not the prior variance of the value per se but of its log scaling.

Description of parameter	Parameter value used for simulations	Prior mean	Prior variance of log scaling
Intrinsic connections *d*_*ij*_ (Hz)	$\left[\frac{4}{5},\ldots ,\frac{1}{5}\right]\cdot 1000$	$\left[\frac{4}{5},\ldots ,\frac{1}{5}\right]\cdot 1000$	$\frac{1}{8}$
Extrinsic connections (Hz)	exp(2) ⋅ 200	200	$\frac{1}{8}$
Rate constants *κ*_*i*_ (Hz)	$\left[\frac{1}{2},\frac{1}{2},\frac{1}{16},\frac{1}{28}\right]\cdot 1000$	$\left[\frac{1}{2},\frac{1}{2},\frac{1}{16},\frac{1}{28}\right]\cdot 1000$	$\frac{1}{16}$
Slope of sigmoid *γ*	$\exp\left(\frac{1}{8}\right)\cdot \frac{2}{3}$	$\frac{2}{3}$	$\frac{1}{32}$
Intrinsic delays *τ* (ms)	1	1	$\frac{1}{32}$
Extrinsic delays *τ* (ms)	4	8	$\frac{1}{32}$
Amplitude of fluctuations	exp(− 2)	1	$\frac{1}{128}$
Exponent of fluctuations	1	1	$\frac{1}{128}$
Amplitude of noise	exp(− 8)	1	$\frac{1}{128}$
Exponent of noise	1	1	$\frac{1}{128}$

**Fig. 4:**
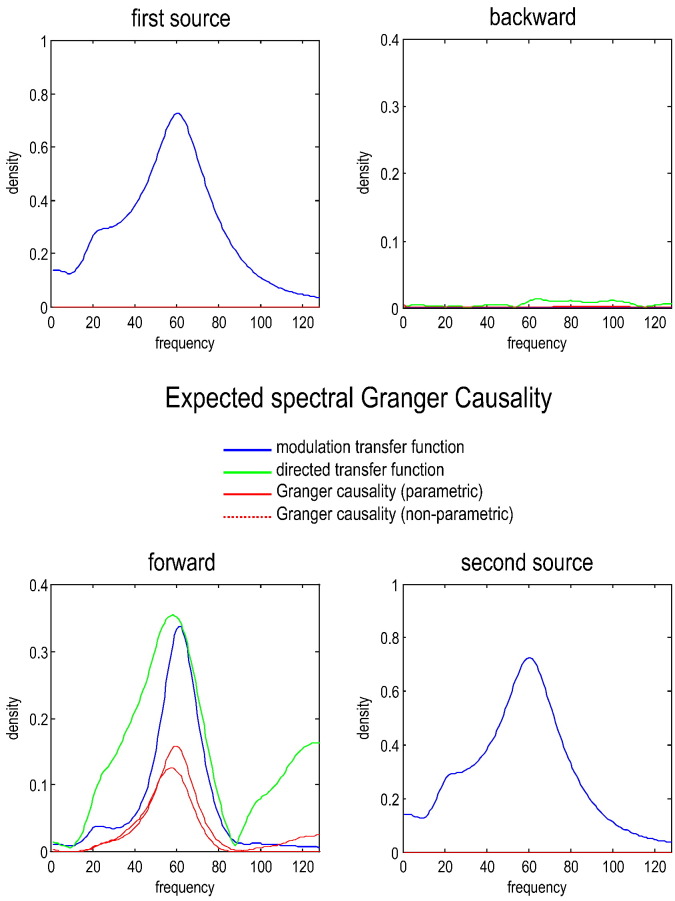
This figure reports the expected modulation transfer functions (blue lines), normalised directed transfer functions (green lines) and the associated spectral Granger causality (red lines: parametric — solid and nonparametric — dotted) under the dynamic causal model shown in Fig. 1. In this example, measurement noise was suppressed (with log-amplitude of − 8). The log-amplitude of the neuronal fluctuations was set at a fairly low level of − 2. These fluctuations had a power law form with an exponent of one. The spectral measures are the expected values, given the model parameters, and correspond to what would be seen with a very large amount of data. Under these conditions, the (expected) directed transfer functions and Granger causality identify the predominance of gamma in the forward connections — and correctly detect that there is no reciprocal or backward connection.

Equipped with the expressions in Table 1, one can now derive the expected transfer functions and spectral Granger causality for any generative process specified in terms of its parameters. Fig. 4 shows the functions expected using the parameters in Table 2. Note that the expected Granger causalities are not estimates — they are analytic reformulations of the underlying causal architecture using the expressions in Table 1. Given the (effective connectivity) parameters of the state-space model, the only thing that we have to specify is the order of the autoregressive model of linear dependencies (we used a model order of *p* = 16 here and throughout).

The expected modulation (blue lines) and (normalised) directed transfer functions (green lines) report the shared variance between the two regions as a function of frequency. For these parameters, the causal spectral measures (directed transfer functions and Granger causality) properly identify the gamma peak and – more importantly – assign all the causal effects to the forward connections — with no Granger causality in the backward direction. Note that the directed transfer functions are normalised and therefore have a different form to the modulation transfer functions. The modulation transfer functions report the total amount of power (at each frequency) in one source that appears in another. In short, under low level measurement noise, Granger causality can properly identify the directed functional connectivity in both qualitative and quantitative terms. In the next section, we explore the parameter space over which Granger causality retains its validity.

## The effects of dynamical instability and measurement noise

**Fig. 5:**
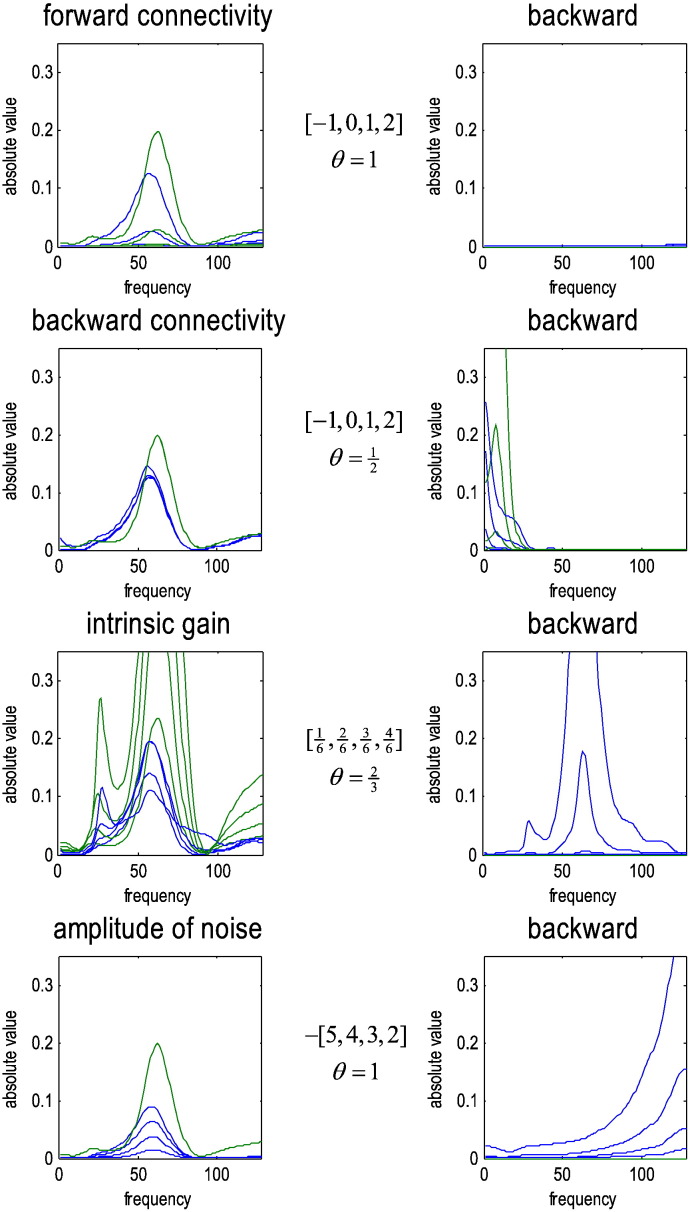
This figure reports the results of repeating the analysis of the previous figure but under different levels of various model parameters. The left column shows the estimates of forward connectivity in terms of the (normalised) modulation transfer function (green lines) and (parametric) spectral Granger causality estimates based upon an AR(16) process (blue lines). The modulation transfer functions were normalised according to Eq. (6) and can be regarded as the ‘true’ Granger causality. The right-hand columns show the equivalent results for the backward connection (which did not exist). The first row shows the effects of increasing the extrinsic forward connection strengths. The ranges of parameters considered are shown as log scaling coefficients (in square brackets) of their expectation (shown below the range and in Table 2). The second, third and fourth rows report the results of similar changes to the backward connection strength, the intrinsic gain (slope of the sigmoid function in Fig. 2) and the amplitude of measurement noise in the second channel. With these parameters, increases in forward connectivity amplify the coupling in the gamma range in the forward direction, while increases in backward effective connectivity are expressed predominantly in the beta range. The key thing to note here is that changes in extrinsic connectivity are reflected in a veridical way by changes in spectral causality — detecting increases in backward connectivity when they are present and not when they are absent. However, Granger causality fails when intrinsic gain and measurement noise are increased — incorrectly detecting strong backward influences that peak in the gamma band high-frequency ranges.

We repeated the above analyses for different levels of some key parameters to illustrate two conditions under which Granger causality may fail. These parameters were the forward and backward extrinsic connection strengths, the intrinsic gain or the slope of the sigmoid function (Kiebel et al., 2007) and the amplitude of measurement noise (in the second source). Table 2 lists the prior expectations for these parameters, while Fig. 5 lists the levels we explored in terms of their log scaling. Fig. 5 shows the results of these analyses in terms of (normalised) modulation transfer functions (green lines) and Granger causality (blue lines) associated with the forward (left panels) and backward connections (right panels). Pleasingly, when increasing forward connection strengths, Granger causality increases in proportion, without detecting any backward Granger causality. Similarly, when the backward effective connectivity was increased, Granger causality detected this, while its estimate of forward influences remained unchanged. Interestingly, under these neural mass model parameters, the forward modulation transfer functions peak in the gamma range, while the backward connections peaked in the lower beta range. This is reminiscent of physiological findings: for example, recent findings suggest that the superficial layers show neuronal synchronization and spike-field coherence predominantly in the gamma frequencies, while deep layers prefer lower (alpha or beta) frequencies (Roopun et al., 2006, Maier et al., 2010, Buffalo et al., 2011). Since feedforward connections originate predominately from superficial layers and backward connections from deep layers, this suggests that forward connections use relatively high frequencies, compared to backward connections (Bosman et al., 2012).

In contrast to changes in extrinsic connectivity, when intrinsic gain and measurement noise were increased, Granger causality fails in the sense that it detects strong and spectrally structured backward Granger causality. Interestingly, for increases in intrinsic gain, this spurious influence was localised to the gamma range of frequencies. In this example, the increase in measurement noise was restricted to the second channel and produced spurious spectral causality measures in high-frequency regimes. We now consider the reasons for these failures in terms of dynamical instability and measurement noise.

### Dynamical instability

The failure of Granger causality with increasing intrinsic connectivity is used to illustrate a key point when modelling dynamical (biophysical) systems with autoregressive models. Autoregression processes model temporal dependencies among observations that are mediated by (long memory) dynamics of hidden states. One can express the dependencies between the current and past states as follows (using a local linear approximation):

1 $$\frac{\partial x\left(t\right)}{\partial x\left(t-\tau \right)}=\exp\left(\tau \cdot \nabla_{x}f\right)$$

Clearly, if one wanted to model these dependencies with a *p*-th order autoregressive process, one would like the dependency above to be negligible when *τ* > *p* (where *p* becomes an interval of time). In other words, the eigenvalues *λ* of the Jacobian ∇_*x*_*f* should be sufficiently small to ensure that:

2 $$\frac{\partial x\left(t\right)}{\partial x\left(t-p\right)}\approx 0\Leftrightarrow \exp\left(p\cdot \nabla_{x}f\right)=U\cdot \exp\left(p\cdot \lambda \right)\cdot U^{-}\approx 0.$$

Here (*U*, *U*^−^) are the right and left eigenvectors of the Jacobian ∇_*x*_*f* = *U* ⋅ *λ* ⋅ *U*^−^ and *λ* is a diagonal matrix containing eigenvalues — whose real parts are negative when the system is stable. This means that the autoregressive characterisation of temporal dependencies may be compromised by long range correlations of the sort associated with slowing near (transcritical) bifurcations. Note that transcritical slowing does not mean that fluctuations or oscillations slow down — it means that modes of fast (e.g., gamma) activity decay slowly, where the rate of decay is determined by the real part of the largest eigenvalue (aka the Lyapunov exponent).

Practically, this suggests that if the (neuronal) system is operating near a transcritical bifurcation — and its eigenvalues approach zero from below, then the autoregressive formulation will not converge and associated spectral (Granger causality) measures become unreliable. Technically, dynamical instability means that the covariance among observations becomes ill-conditioned, where the cross covariance (at zero lag) can be expressed in terms of the (real part of the) eigenvalues as follows:

3 Σ0=−12∇xg⋅UReλ−1U−ΣvUU−⋅∇xgT+Σ0w.

Eq. (3) means that the mode or pattern of activity described by the eigenvector whose eigenvalue approaches zero will decay much more slowly than the other modes and will dominate the cross-covariance. This is important because the auto regression coefficients are computed using the inverse of the cross-covariance matrix $a=C^{-1}\tilde{\Sigma }$. As the largest (real) eigenvalue approaches zero, the inverse cross covariance matrix therefore becomes singular, precluding a unique solution for the autoregression coefficients.

**Fig. 6:**
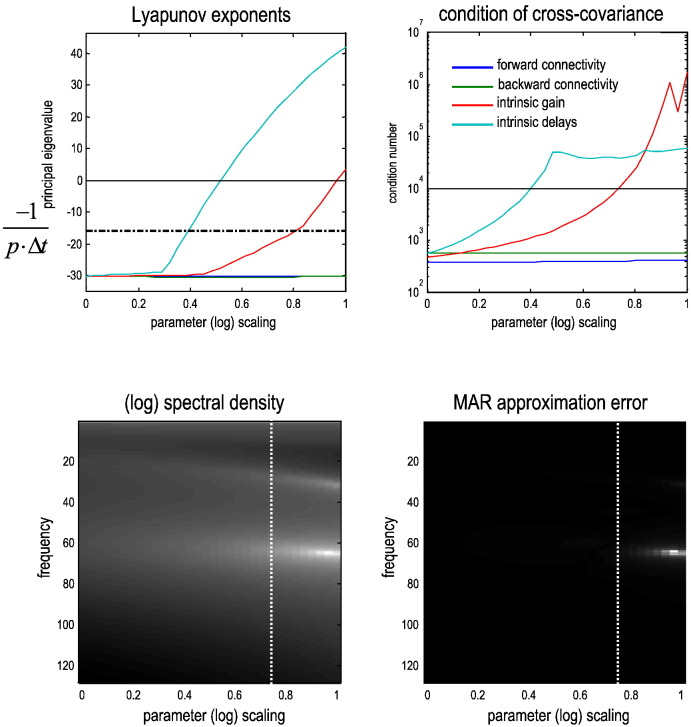
This figure shows why Granger causality based upon (finite-order) autoregressive processes fail under increasing intrinsic gain (and any other parameter that induces instability through a transcritical bifurcations). *Upper left panel*: this show the principal (largest real part of the) eigenvalue of the systems Jacobian; also known as the Lyapunov exponent. When this eigenvalue approaches zero from below, perturbations of the associated eigenfunction of hidden states decay very slowly — and become unstable when the eigenvalue becomes positive. One can see that increasing the intrinsic gain (red line) induces a transcritical bifurcation at about a log scaling of one. Furthermore, at a log scaling of .8, the time constant associated with the eigenvalue becomes greater than $p\cdot \Delta t=\frac{16}{256}=\frac{1}{16}$ seconds (dashed line). The blue and green lines show the equivalent results as the (forward and backward) extrinsic connectivity is increased — showing no effect on the eigenvalue. However, increasing the intrinsic delay induces instability and critical slowing. This causes the condition number of the cross covariance matrix to increase, where a large condition number indicates a matrix is (nearly) singular or rank efficient. *Upper right panel*: this shows the corresponding condition number of the cross covariance matrix used to compute the autoregression coefficients, using the same format as the previous panel. *Lower left panel*: this shows the corresponding (log) spectral density (of the first source) over the same range of intrinsic gains shown in the upper panel. It shows that the beta and gamma peaks increase in frequency and amplitude with intrinsic gain. *Lower right panel*: this shows the difference between the expected auto spectrum (shown on the right) and the approximation based upon autoregression coefficients estimated using the associated cross-covariance functions. It can be seen that these differences become marked when the condition number exceeds about 10,000.

Based on this heuristic analysis, one might anticipate that the extrinsic connectivity parameters do not increase the principal eigenvalue of the Jacobian (also known as the local Lyapunov exponent), whereas the intrinsic gain parameter does. Fig. 6 (upper left panel) shows that increasing the intrinsic gain (expressed in terms of log scaling) produces eigenvalues with associated time constants of over 1/*λ* ≥ 1/16 s — which exceeds the temporal support of an AR(16) model with 1/256 second time bins. The upper right panel shows the condition number (the ratio of the largest eigenvalue to the smallest) of the cross-covariance matrix, where a larger condition number indicates a (nearly) singular matrix. The lower panels of Fig. 6 illustrate the ensuing failure of an autoregressive characterisation of spectral responses by comparing the expected spectrum (from the first source) with the autoregressive approximation associated with the coefficients derived analytically from the expected cross-covariance function. One can see that marked differences are evident when the condition number of the cross covariance matrix exceeds about 10,000. Clearly, one could consider increasing the order of the autoregressive process; however, when the cross covariance matrix becomes (nearly) singular, one would need a (nearly) infinite order process.

Other model parameters that cause the principal eigenvalue to approach zero from below include the intrinsic and extrinsic delays (see Fig. 6). This is important, because neuronal systems necessarily have delays, which effectively increases the number of hidden states to infinity: strictly speaking, the introduction of delays into the system's characteristic function induces an infinite number of eigenvalues (Driver, 1977). This (almost) inevitably produces near zero Lyapunov exponents and is an important source of critical behaviour in biological systems (Jirsa and Ding, 2004). Another perspective on the failure of autoregressive formulations is based on the fact that a (linear) state-space model with *m* hidden states has an AR(*p*) formulation, where *m* = 2*p* (Nalatore et al., 2007). When the number of effective states increases to infinity, the associated infinite-order AR process cannot be approximated with a finite-order autoregressive process.

**Fig. 7:**
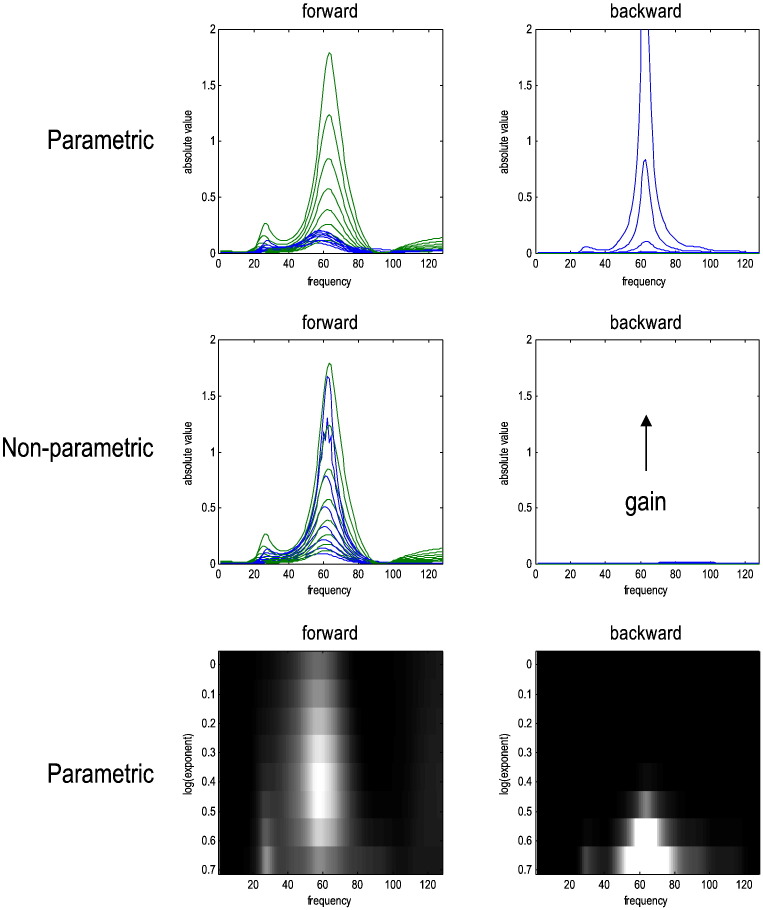
This figure presents a more detailed analysis of the effects of increasing intrinsic gain on spectral Granger causality measures. The left column shows the (normalised) modulation transfer function (green line) and Granger causality (blue line) over eight (log) scaling values of intrinsic connectivity. The right panels show the equivalent results for the backward connection. The top row shows the expected parametric Granger estimates based upon an autoregressive process, while the middle row shows the equivalent results for the expected nonparametric measure. The lower row shows the same results as in the upper row but in image format (with arbitrary colour scaling) to clarify the effects of increasing intrinsic gain. The key thing to take from these results is that parametric Granger causality is unable to model the long-range correlations induced by dynamical instability and, improperly, infers a strong backward connectivity in a limited gamma range. In contrast, the nonparametric measure is not constrained to model autoregressive dependencies and properly reflects the increase in forward coupling — without reporting any backward coupling.

One remedy for systems that show critical behaviour is to abandon the finite-order autoregressive formulation and consider nonparametric estimates of Granger causality based on spectral matrix factorisation (Nedungadi et al., 2009). Fig. 7 takes a closer look at the effect of increasing intrinsic gain and compares the performance of autoregressive and Wilson–Burg spectral causality measures. As one might predict, the Wilson–Burg estimates finesse the problem of unstable modes and may therefore be preferable in the characterisation of neuronal timeseries. Heuristically, the Wilson–Burg estimates can do this because they have more degrees of freedom to model (minimum phase) linear dependencies. In other words, minimum phase filters require more numbers to specify them than the number of coefficients available to AR models of the order used. Clearly, this latitude introduces a potential trade-off in terms of overfitting; however, in this setting Wilson–Burg procedures provide better estimates of the true (expected) Granger causality (green lines in Fig. 7).

### Measurement noise

The failure of Granger causality in the context of measurement noise is slightly more problematic. This failure is almost self-evident from the relationship between the modulation transfer function and the directed transfer function. From Table 1:

4 $$g\left(\omega \right)=K\left(\omega \right)\cdot g_{v}\left(\omega \right)\cdot K{\left(\omega \right)}^{†}+g_{w}\left(\omega \right)\propto S\left(\omega \right)\cdot \Sigma_{z}\cdot S{\left(\omega \right)}^{†}.$$

This expression says that the observed spectral density can be decomposed into measurement noise and shared variance mediated by (neuronal) fluctuations. It is the latter that underlies spectral measures of directed functional connectivity — and not the former. We therefore require the directed transfer functions to be proportional to the modulation transfer functions

5 $$K\left(\omega \right)\cdot g_{v}\left(\omega \right)\cdot K{\left(\omega \right)}^{†}\propto S\left(\omega \right)\cdot \Sigma_{z}\cdot S{\left(\omega \right)}^{†}.$$

In this case, the (off-diagonal terms of the) normalised directed transfer functions report the shared variance. This constraint portends some good news and some bad news: the good news is that there is no requirement that the spectral power of the innovations has to be the same for all frequencies (this is why the covariance of the innovations *Σ*_*z*_ ∝ *g*_*z*_(*ω*) is used as a proxy for their spectral density in Table 1 — and why some equalities are proportionalities). This means that normalised spectral measures – like directed transfer functions and Granger causality – do not have to assume the innovations are white. In other words, they can cope with serially correlated innovations *g*_*z*_(*ω*) of the sort we have used for the underlying fluctuations *g*_*v*_(*ω*).

The bad news is that for the proportionality above to hold the measurement noise has to be negligible (or proportional to the shared component). This is a simple but fundamental observation, which means that Granger causality can become an unreliable measure in the presence of substantial measurement noise. This problem was identified nearly half a century ago (Newbold, 1978) and has recently started to attract serious attention: see (Nalatore et al., 2007) for a comprehensive deconstruction of the problem in the context of autoregressive formulations. See also Solo (2007) who consider the effects of measurement noise under AR models and conclude that “state space or vector ARMA models are needed instead.”

In brief, we can summarise the situation as follows: the spectral causality we seek is mediated by the modulation transfer functions. These modulation transfer functions can be approximated by directed transfer functions when measurement noise can be ignored. In this case, the fluctuations and innovations become the same. Furthermore, if we make the simplifying assumption that the spectral profile of the fluctuations (and implicitly the innovations) is the same for all sources, we can replace their cross spectral density with their covariance. This can be expressed formally to recover the conventional expression for spectral Granger causality:

6 $$\begin{array}{c} G_{\mathrm{ij}}\left(\omega \right)=-\ln\left(1-\left(g_{\mathrm{vjj}}\left(\omega \right)-\frac{{\left|g_{\mathrm{vij}}\left(\omega \right)\right|}^{2}}{g_{\mathrm{vii}}\left(\omega \right)}\right)\frac{{\left|K_{\mathrm{ij}}\left(\omega \right)\right|}^{2}}{K_{\mathrm{ii}}\left(\omega \right)}\right)\\ =-\ln\left(1-\left(g_{\mathrm{zjj}}\left(\omega \right)-\frac{{\left|g_{\mathrm{zij}}\left(\omega \right)\right|}^{2}}{g_{\mathrm{zii}}\left(\omega \right)}\right)\frac{{\left|S_{\mathrm{ij}}\left(\omega \right)\right|}^{2}}{S_{\mathrm{ii}}\left(\omega \right)}\right)\;\mathrm{when}\;g_{w}\left(\omega \right)=0:\forall \omega \\ =-\ln\left(1-\left(\Sigma_{\mathrm{zjj}}-\frac{\Sigma_{\mathrm{zij}}^{2}}{\Sigma_{\mathrm{zii}}}\right)\frac{{\left|S_{\mathrm{ij}}\left(\omega \right)\right|}^{2}}{g_{\mathrm{ii}}\left(\omega \right)}\right)\;\mathrm{when}\;g_{w}\left(\omega \right)=0\;\mathrm{and}\;g_{v}\left(\omega \right)\propto \Sigma_{z}:\forall \omega . \end{array}$$

**Fig. 8:**
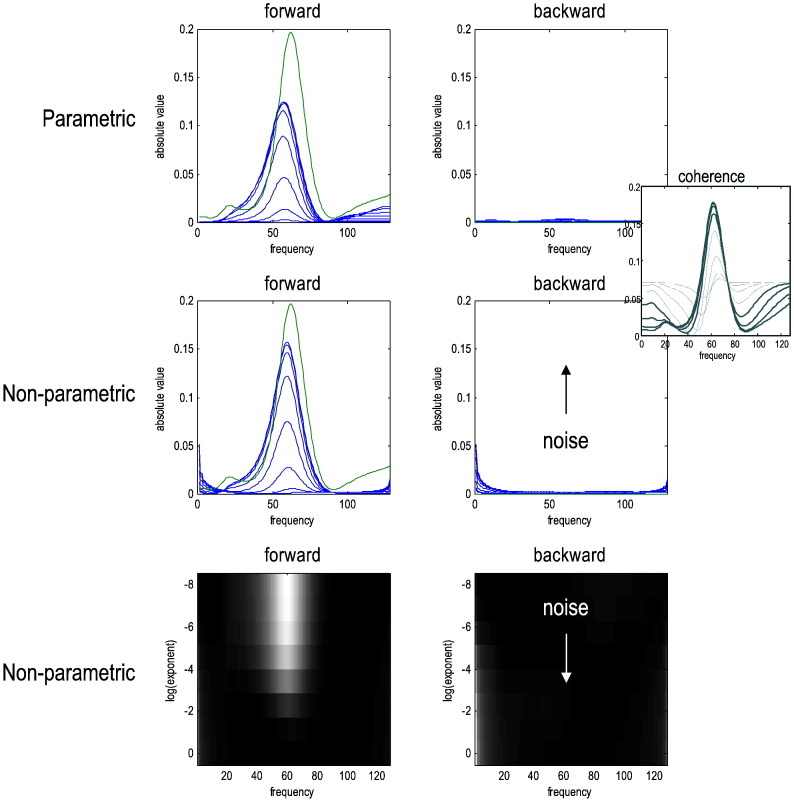
This figure uses the same format as in the previous figure; however here, we have increased the amplitude of measurement noise (from a log amplitude − 8 to − 2). This measurement noise had channel-specific and shared components at a log ratio of one (i.e., a ratio of about 2.72). At nontrivial levels of noise (with a log-amplitude of about − 4) the expected Granger causality fails for both parametric and nonparametric measures. The predominant failure is a spurious reduction in the forward spectral causality and the emergence of low-frequency backward spectral causality with nonparametric measures. The inset on the upper right shows the impact of noise on the coherence between the two channels at low (solid) and high (dotted) levels of noise.

Fig. 8 shows what happens when measurement noise cannot be ignored by increasing its log-amplitude from trivial values (of − 8) to the level of the fluctuations. Here, we increased both the power of the measurement noise that was shared and unique to each channel (where the shared component was smaller than the unique component by a log-amplitude of one). This simulates a range of signal-to-noise ratios from almost negligible to very high levels. This can be seen in the inset of Fig. 8, which shows the coherence between the two channels for the eight noise levels considered. For the first four levels, nearly all the coherence is mediated by neuronal fluctuations; whereas at the last level of measurement, noise dominates the coherence (through the shared component).

The effect of noise, as one might intuit, starts to emerge when the noise is no longer trivial in relation to signal — here at about a log-amplitude of − 4. At this point, the Granger causal measures of forward connectivity start to fall and nontrivial backward connectivity emerges. Interestingly, in the nonparametric case, the spurious spectral coupling is in the same (low-frequency) ranges for both forward and backward connections. One might suppose that this is a reflection of the symmetrical cross spectral density induced by noise.

It should be emphasised that the levels of noise that impact on spectral causality measures are large in relation to typical electrophysiological recordings, especially LFP recordings. In typical data analysis situations, one should be able to diagnose recordings with high levels of shared measurement noise using measures that are sensitive to non-causal shared variance (e.g., volume conduction), like imaginary coherence and the weighted phase-locking index (Nolte et al., 2004, Vinck et al., 2012). In these situations, it is well-known that Granger causal analysis can be confounded (Nalatore et al., 2007). In the next section, we explore how DCM can mitigate this problem.

In summary, the naive application of Granger causality to measured data is unreliable unless the measurements are relatively noiseless. This observation speaks to the fundamental difference between approaches that try to characterise dependencies among observations and approaches that acknowledge observations are generated by hidden states, such as dynamic causal modelling. Does this mean that spectral Granger causality should be abandoned in the setting of noisy measurements? Not necessarily. In the final section, we consider how valid Granger causality estimates can be recovered from dynamic causal modelling.

## Dynamic causal modelling of Granger causality

In the previous section, we saw that nonparametric Granger causality finesses the problems associated with characterising biological timeseries generated by coupled and delayed dynamics with unstable (slow) modes of behaviour. However, nonparametric Granger causality fails in the presence of measurement noise. Here, we provide a proof of principle that the problem of measurement noise can be dissolved by computing the Granger causality based upon the modulation transfer functions estimated by dynamic causal modelling (see Eq. (6)).

**Fig. 9:**
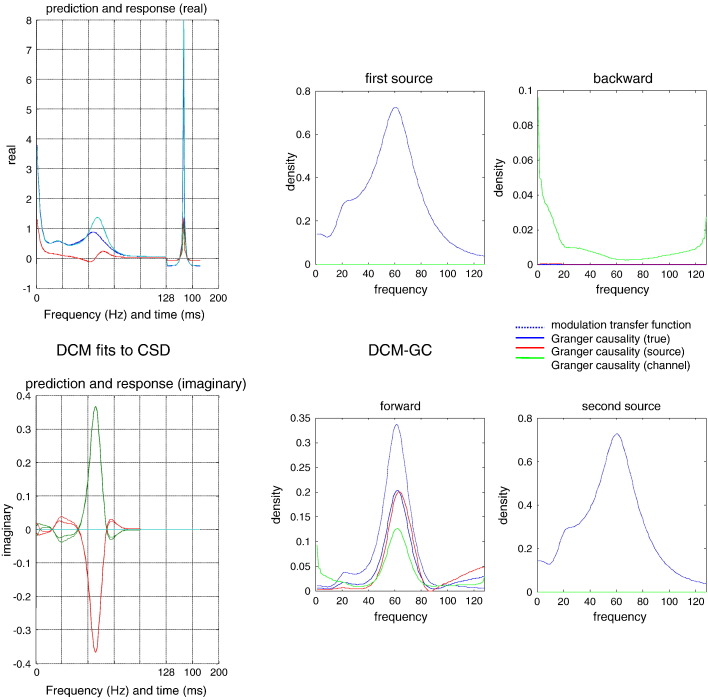
This figure reports the results of a Granger causality analysis that finesses the measurement noise problem by basing spectral causality measures on the parameters estimated by dynamic causal modelling: *Left panel*: these plots show the observed (full lines) and predicted (dotted lines) cross spectra, in terms of real (upper panel) and imaginary (lower panel) parts. In most regimes, the fit is almost perfect; however, there are some small prediction errors around 20 Hz in the imaginary part. The first portion of these predicted and observed profiles corresponds to the spectra, while the last portion is the (real) cross-covariance function. Both of these data features are used to improve the convergence of model inversion. *Right panel*: this shows the results of Granger causality measures based upon DCM using the format of Fig. 4. In this instance, the modulation transfer function is a maximum a posteriori estimate (dotted line). The solid blue line is the normalised modulation transfer function based on the true parameter values and can be regarded as the true Granger causality (see Eq. (6)). Crucially, the Granger causality among the sources (red line) correctly reports the absence of any backward coupling and is almost identical to the true Granger causality. Contrast this with the naive Granger causality (green line) based on observed responses with measurement noise. Here, the backward Granger causality attains nontrivial levels at low frequencies that are not present.

Put simply, using an explicit model of (realistic) fluctuations and measurement noise, one can estimate the Granger causality that would have been seen in the absence of noise. This is illustrated in Fig. 9, where we have inverted a model of the generative process using standard (variational Laplace) procedures (Friston et al., 2007) to estimate the effective connectivity parameters. These parameters provide the modulation transfer functions — and the corresponding Granger causality measures that properly reflect the spectral structure of forward influences and the absence of backward connectivity. In this example, we fitted the expected cross spectra in channel space using the (known) form of the neural mass model with (unknown) parameters and the usual priors for this model (provided in Table 2).

For those people not familiar with dynamic causal modelling, DCM is a Bayesian model comparison and inversion framework for state-space models formulated in continuous time. It uses standard variational Bayesian procedures to fit timeseries or cross spectra – under model complexity constraints – to provide *maximum a posteriori* estimates of the underlying (effective connectivity) model parameters. See Friston et al. (2012) for more details in this particular setting. This iterative procedure usually converges within about 32 iterations, producing the sort of fits shown in the left panels of Fig. 9. In short, DCM solves the inverse problem of recovering plausible parameters (of both neuronal dynamics and noise) that explain observed cross spectra.

In summary, provided that one can solve the inverse problem posed by dynamic causal modelling, one can recover veridical Granger causality measures in the spectral domain. Crucially, this requires accurate models of the underlying generative process. From the point of view of dynamic causal modelling, these measures provide an intuitive and quantitative report of the directed frequency-specific influences mediated by effective connectivity. Specifically, they are the normalised modulation transfer functions (as opposed to the normalised directed transfer functions). From the point of view of spectral Granger causality, DCM has just been used to place constraints on the parametric form of the underlying dynamics, which enable observed power to be partitioned into signal (generated by hidden states) and noise.

## Conclusion

In conclusion, we have shown that naïve Granger causality is not appropriate for noisy measurements of coupled dynamical systems with delays. Having said this, it is fairly straightforward to compute Granger causality measures of directed functional connectivity from estimates of directed effective connectivity — as provided by dynamic causal modelling. In their analysis of measurement noise, Nalatore et al. (2007) proposed a solution based upon a linear state-space model and Bayesian model inversion (an expectation maximisation scheme based upon Kalman smoothing). The same approach – to estimating measurement noise by modelling dependencies with linear state-space models – appears to have been proposed independently by Sommerlade et al. (2012). Although this approach may fail when the process generating data exhibits critical dynamics, it nicely highlights the need to explicitly model hidden states generating observed data — such that covariance induced by fluctuations in hidden states can be separated from covariance due to measurement noise; see also Robinson et al. (2004). In short, a failure to model hidden states may produce false inferences when naïvely applying spectral Granger causality to observed data.

This conclusion shifts the problem from worrying about the shortcomings of Granger causality to worrying about the problems posed by dynamic causal modelling. These problems should not be underemphasised (Daunizeau et al., 2011): in the illustration above, we used a dynamic causal model that had the same form as the process generating noisy observations. Clearly, this model will not be known in real world applications. This means that the DCM has to be optimised in relation to the data at hand — using Bayesian model comparison or averaging (Penny et al., 2004). This speaks to an open issue in DCM; namely, how to score and invert alternative models of observed data in an accurate and efficient fashion. In one sense, this problem is the focus of nearly all current work on dynamic causal modelling and – although much progress has been made – dynamic causal modelling is still in its infancy. This model optimisation speaks to the continuous process of perfecting the underlying model of neuronal dynamics as more information about neuronal circuits becomes available. This is at the heart of a Bayesian approach to data modelling: placing knowledge in the model to provide more informed constraints and better estimates of causal interactions.

We have distinguished between estimates of Granger spectral causality based upon autoregressive processes and spectral factorisation as parametric and nonparametric respectively (Dhamala et al., 2008). For some people, this is contentious because both rest upon models of statistical dependencies that have implicit parameters. For example, the autoregression coefficients in autoregressive models or the minimum phase filters produced by spectral factorisation. In this sense, both are parametric. Conversely, unlike dynamic causal modelling, neither parametric nor nonparametric Granger causality are equipped with parameterised models of how dependencies are generated. In this sense, they are both nonparametric. The slightly unfortunate use of parametric and nonparametric could be finessed by explicit reference to the underlying model of dependencies; e.g., autoregressive or minimum phase (as suggested by our reviewers).

It is well-known that long-range temporal autocorrelations are problematic for Granger causality. Non-invertible (even if causal) filtering can induce such autocorrelations; as will long-memory processes (e.g. power–law autocorrelation decay found in fractionally-integrated autoregressive processes). Granger causality is not well-defined for such processes because they do not satisfy prerequisite spectral conditions (Geweke, 1982). We have considered this issue in terms of dynamical instability in the vicinity of transcritical bifurcations — when the system's principal Lyapunov exponent (real eigenvalue) approaches zero from below. In spectral theory, this instability can be characterised in terms of the *spectral radius*; which is the largest (supremum) over a system's spectrum of absolute eigenvalues. A recent discussion of these issues can be found in Barnett and Seth (2014).

The focus of this technical note has been rather pragmatic: it has focused on the technical issue of estimating Granger causality in the presence of measurement noise and long range correlations. Furthermore, we have restricted our treatment to spectral measures of Granger causality. One might ask why measure Granger causality with dynamic causal modelling if one has already estimated the causal influences (effective connectivity and associated transfer functions) en route. In the setting of effective and functional connectivity, Granger causality has been cast as a measure of directed functional connectivity (Friston et al., 2013). Given that functional connectivity is a measure of statistical dependencies or mutual information, this means that Granger causality measures the directed mutual information between the present state (of a target) and the past state (of a source). Formally, directed mutual information corresponds to transfer entropy (Lindner et al., 2011). This is important because there is equivalence between Granger causality and transfer entropy (Barnett et al., 2009), which therefore allows one to quantify directed information transfer in terms of Granger causal estimates. This suggests that it is possible to relate causal influences (effective connections) to their information theoretic consequences in a quantitative sense.

Finally, we have not addressed how to assess the significance of Granger causality. Generally, this would proceed using some form of nonparametric inference based upon (surrogate) data in which directed temporal dependencies are destroyed. Although our focus has been on estimation, as opposed to inference, it is worth noting that – from the perspective of DCM – inference rests on comparing the evidence for models generating Granger causality metrics. In other words, one would first select the DCM with the highest model evidence, after which Granger causal measures would be used to characterise directed functional connectivity in a quantitative fashion.

## Figures and Tables

**Table:** 

Variable	Description
*x*(*t*)	Hidden states of a state-space or dynamic causal model
*y*(*t*)	Observed measurements or response variable
*f*(*x*, *θ*)	Nonlinear equations of motion or flow
*g*(*x*, *θ*)	Nonlinear observation or measurement equation
*v*(*t*)	Random fluctuations in the motion of hidden states
*w*(*t*)	Random measurement noise
*z*(*t*)	Innovations of an autoregressive model
*Σ*_*v*_(*τ*, *θ*)	Covariance function of fluctuations
*Σ*_*w*_(*τ*, *θ*)	Covariance function of noise
*Σ*_*z*_ ∝ *ψ*(0) ⋅ *ψ*(0)*	Covariance matrix of innovations
∇_*x*_*f*	Jacobian or gradient of flow with respect to hidden states
*k*(*τ*)	First order (Volterra) kernel function of lag
*Σ*(*τ*)	Covariance function of measurements
*C*	Covariance matrix of measurements
$\tilde{\Sigma }$	Lagged covariance matrix of measurements
*Y*(*ω*) = *S*(*ω*) ⋅ *Z*(*ω*)	Fourier transform of measurements
*S*(*ω*)	Directed transfer function from innovations to measurements
*K*(*ω*) = **F**[*k*(*τ*)]	Modulation transfer function from fluctuations to measurements
*A*(*ω*) = **F**[*a*]	Discrete Fourier transform of autoregression coefficients
*Z*(*ω*) = **F**[*z*(*t*)]	Fourier transform of innovations
*g*(*ω*) = **F**[*Σ*(*τ*)]	Spectral density of measurements
*g*_*v*_(*ω*) = **F**[*Σ*_*v*_(*τ*)]	Spectral density of fluctuations
*g*_*w*_(*ω*) = **F**[*Σ*_*w*_(*τ*)]	Spectral density of noise
*Ψ*(*ω*) = **F**[*ψ*(*τ*)]	Minimum phase spectral factors of measurements
*ψ*(*τ*)	First-order (causal) kernels associated with spectral factors

*Glossary of variables*: this glossary provides a brief description of the variables used in Table 1 and the main text.

## Appendix A

This appendix describes the integration of delay differential equations using a high order Taylor expansion in generalised coordinates of motion. This scheme was used to generate the simulated timeseries and their expected cross spectra shown in Fig. 3 — and to evaluate the Jacobian and associated transfer functions that determine the expected Granger causality in subsequent analyses. The Jacobian depends sensitively on delays as described below.

Consider the problem of integrating (solving) the set of delay differential equations where *τ*_*ij*_ is the (asymmetric) delay from state *j* to *i*

A.1 $${\dot{x}}_{i}\left(t\right)=\sum_{j}f_{i}\left(x_{j}\left(t-\tau_{\mathrm{ij}}\right)\right).$$

Using a local linear approximation and Taylor's theorem, we have

A.2 $${\dot{x}}_{i}\left(t\right)=\sum_{j}\frac{\partial f_{i}}{\partial x_{j}}x_{j}\left(t-\tau_{\mathrm{ij}}\right)=\sum_{j}\frac{\partial f_{i}}{\partial x_{j}}\left(x_{j}\left(t\right)+{\dot{x}}_{j}\left(t\right)\left(-\tau_{\mathrm{ij}}\right)+\frac{1}{2}{\ddot{x}}_{j}\left(t\right){\left(-\tau_{\mathrm{ij}}\right)}^{2}+\ldots \right).$$

This can be expressed more compactly in matrix form using *J* = ∇_*x*_*f* (evaluated at the current state or fixed point) and the Hadamard product ×:

A.3 $$\dot{x}=\mathrm{Jx}+\left(J\times \left(-\tau \right)\right)\dot{x}+\frac{1}{2}\left(J\times \left(-\tau \right)\times \left(-\tau \right)\right)\ddot{x}+\ldots$$

If we now assume the existence of a (first-order) approximation of Eq. (A.3) with the form $\dot{x}\approx \mathrm{Qx}$, then substitution into Eq. (A.3) gives

A.4 $$\dot{x}\approx \mathrm{Jx}+\left(J\times \left(-\tau \right)\right)\mathrm{Qx}+\frac{1}{2}\left(J\times \left(-\tau \right)\times \left(-\tau \right)\right)Q^{2}x+\ldots$$

This means that the (approximate) Jacobian we seek is the solution to the matrix polynomial:

A.5 $$Q=J+\left(J\times \left(-\tau \right)\right)Q+\frac{1}{2}\left(J\times \left(-\tau \right)\times \left(-\tau \right)\right)Q^{2}+\ldots$$

This polynomial can be solved reasonably efficiently using the Robbins Monro algorithm as follows (using a suitably large *N* that ensures convergence of the Taylor expansion).

A.6 $$Q_{i+1}=\frac{1}{2}Q_{i}+\frac{1}{2}\sum_{n=0}^{N}\frac{1}{n!}\left(J\times \left(-\tau \right)\times \left(-\tau \right)\times \ldots \times \left(-\tau \right)\right)Q_{i}^{n}.$$

The algorithm can be initialised with the solution to the first-order approximation to Eq. (A.5) (this approximation is used directly for simpler neural mass models):

A.7 $$\begin{array}{c} Q_{o}=J-\left(J\times \tau \right)Q_{o}\\ ={\left(I+J\times \tau \right)}^{-1}J. \end{array}$$

This scheme appears to give good approximations when checked numerically.

## Footnotes

1 Please see later for a discussion of the terms parametric and nonparametric in this context.
